# Sarand: exploring antimicrobial resistance gene neighbourhoods in complex metagenomic assembly graphs

**DOI:** 10.1093/nargab/lqag066

**Published:** 2026-07-14

**Authors:** Somayeh Kafaie, Shahlla Naseri, David B J Mahoney, Travis Gagie, Robert G Beiko, Finlay Maguire

**Affiliations:** Department of Mathematics and Computer Science, Saint Mary’s University, 912 Robie Street, Halifax, Nova Scotia B3H 3C3, Canada; Faculty of Computer Science, Dalhousie University, 6050 University Avenue, Halifax, Nova Scotia B3H 4R2, Canada; Department of Mathematics and Computer Science, Saint Mary’s University, 912 Robie Street, Halifax, Nova Scotia B3H 3C3, Canada; Faculty of Computer Science, Dalhousie University, 6050 University Avenue, Halifax, Nova Scotia B3H 4R2, Canada; Faculty of Computer Science, Dalhousie University, 6050 University Avenue, Halifax, Nova Scotia B3H 4R2, Canada; Department of Community Health and Epidemiology, Dalhousie University, 5790 University Avenue, Halifax, Nova Scotia B3H 1V7, Canada; Faculty of Computer Science, Dalhousie University, 6050 University Avenue, Halifax, Nova Scotia B3H 4R2, Canada; Faculty of Computer Science, Dalhousie University, 6050 University Avenue, Halifax, Nova Scotia B3H 4R2, Canada; Faculty of Computer Science, Dalhousie University, 6050 University Avenue, Halifax, Nova Scotia B3H 4R2, Canada; Department of Community Health and Epidemiology, Dalhousie University, 5790 University Avenue, Halifax, Nova Scotia B3H 1V7, Canada

## Abstract

Antimicrobial resistance (AMR) is a major global challenge to human and animal health. The genomic element (e.g. chromosome, plasmid, and genomic islands) and neighbouring genes associated with an AMR gene play a major role in its function, regulation, evolution, and propensity to undergo lateral gene transfer. Therefore, characterizing these genomic contexts is vital for effective AMR surveillance, risk assessment, and stewardship. Metagenomic sequencing is widely used to identify AMR genes in microbial communities but fragmentary short-read data do not directly provide this critical contextual information. Assembly of these reads provides some contextual information but fails to recover many mobile genetic elements. Here, we introduce Sarand, a method retaining some of the sensitivity of read-based methods while providing the genomic context of assembly by extracting AMR genes and their associated context directly from metagenomic assembly graphs. Sarand uses BLAST-based homology searches with coverage statistics to identify and visualize AMR gene contexts while filtering false chimeric contexts. Using both real and simulated metagenomic data, we show that Sarand outperforms metagenomic assembly and other recently developed graph-based tools in terms of precision and sensitivity for this problem. Sarand enables effective extraction of metagenomic AMR gene contexts to better characterize AMR evolutionary dynamics within complex microbial communities.

## Introduction

Antimicrobial resistance (AMR) is a major global health threat [1] with an estimated 1.27 million deaths attributed to AMR in 2019 alone [2]. Lateral gene transfer (LGT) of mobile genetic elements (MGEs), such as plasmids and genomic islands, can drive the spread of AMR between organisms and habitats [3]. For example, the dynamics of colistin resistance among livestock-associated microbial communities influence the dynamics of colistin resistance in clinically associated pathogens [4]. To develop effective interventions for AMR in this intersectoral ‘One Health’ context, we need to be able to characterize the evolution and transmission of AMR within and between microbial communities. An AMR gene’s associated genomic element(s) (e.g. chromosome, plasmid, or genomic island) and neighbouring genes can play key roles in its function [5], regulation [6, 7], evolution [8], and likelihood of undergoing LGT [9, 10]. Determining what constitutes a biologically relevant and conserved gene neighbourhood is still a broad and open area of research (e.g. [11–15]). For example, Koonin and colleagues [15–17] have found that neighbourhoods are rarely structurally conserved, even in closely related bacteria, beyond scales of 2–5 genes. However, even arbitrary/partial AMR gene neighbourhoods have been effectively used to analyse the dynamics of carbapenemase dissemination in *Klebsiella pneumoniae* [18] and characterize the global sewage resistome [19]. Therefore, to improve AMR surveillance and research, we need effective computational approaches to systematically explore the genomic neighbourhood of AMR genes across key microbial ecosystems such as human and animal microbiomes, agricultural soil, and wastewater.

The isolation and sequencing of individual genomes from these communities can provide an inventory of AMR genes and their contexts. However, even with advances in single-cell methods [20] and culturing approaches [21, 22], this is only feasible for a subset of microbes in most host-associated or environmental communities. Alternatively, metagenomics, in which most DNA present in a community is simultaneously sampled and sequenced, offers a powerful tool to characterize complex microbial communities.

Although long-read sequencing technologies such as Oxford Nanopore Technologies [23] and Pacific BioSciences [24] platforms are now used for metagenomic sequencing [25, 26], the greater throughput of relatively short-read technologies (150–300 bp) means they still comprise the majority of metagenomic datasets. As these reads are shorter than most genes, it is rarely possible to identify specific AMR alleles or recover the genomic context directly from these reads.

Metagenomic assembly, in which shared sequences between reads are used to recover longer sequences, offers one possible solution to this problem. Assembly algorithms aim to construct graphs that represent candidate orderings of sequence reads (or their constituent *k*-mers), then condense these graphs to produce unambiguous contiguous sequences (*contigs*). Sequencing errors, repetitive DNA segments, and other structural variants make even assembly of individual genomes from pure cultures a challenging task [27], and the MGEs that often bear AMR genes are among the most difficult parts of genomes to correctly assemble [28]. Metagenomic assembly is even more complicated, with the uneven abundance of organisms in a sample, genome differences at the strain level, and nearly identical genome segments across closely related species leading to substantial uncertainty and ambiguity during assembly [27]. This often leads to metagenomic assembly recovering missing, partial, or incorrect gene neighbourhoods around target AMR genes [28, 29].

Direct exploration of the sequence graph generated during metagenomic assembly (before it is condensed into linear contigs) offers an alternative and potentially more sensitive approach to identify and explore the genomic context of key genes within microbial communities [30]. The promise of this approach has been shown in related work resolving closely related strains [31], variant calling [32], and performing rapid gene homology searches [33] in complex metagenomes.

Tools for graph-based genomic context analyses can take a localized assembly approach (such as the MetaCherchant [34] or the earlier protein-based approaches GRASP [35] and HMM-GRASPx [36]) in which reads and *k*-mers related to the query gene(s) are identified and then a local de Bruijn graph constructed representing the query neighbourhood. However, while effective for capturing the metagenomic diversity of the query genes themselves, these localized assembly approaches may be limited in their ability to capture the wider gene neighbourhood(s). Alternatively, other methods first construct the entire assembly graph and then extract the query neighbourhood either manually through scaffolding [30] or through automated graph-theoretic approaches to subgraph extraction, such as Spacegraphcats [37, 38]. However, these extracted subgraphs contain many possible upstream and downstream paths and lack an easy approach to resolve the true genomic neighbourhoods (i.e. correctly join upstream and downstream paths) from false chimeric path pairs that do not exist in any underlying genomes. This problem is particularly acute for mobile AMR genes as they may have multiple genomic contexts within a metagenome and be associated with hard-to-assemble repetitive sequences.

To try and address these challenges, we developed Sarand (https://github.com/beiko-lab/sarand), a new method to extract the genomic neighbourhoods of AMR genes from metagenomic assembly graphs. Sarand uses BLAST-based homology searches to identify AMR gene-associated nodes in the assembly graph, enumerates local upstream and downstream paths, and then applies depth of coverage-based statistics to filter out likely chimeric neighbourhoods. To enhance exploration and comparison of AMR genes within sequenced microbial communities, Sarand also annotates and visualizes the extracted gene neighbourhoods. We evaluated Sarand’s ability to reconstruct AMR gene neighbourhoods compared with metagenomic assembly, local assembly-based MetaCherchant [34], and the subgraph-based Spacegraphcats [37] using low-, medium-, and high-diversity simulated metagenomes as well as 3 real urban sewage metagenomes [39]. These analyses show that Sarand had superior sensitivity ($47\%$ and $134\%$ average improvement over assembled contigs and MetaCherchant, respectively) in identifying AMR gene neighbourhoods while also outperforming other approaches in avoiding chimeric/false neighbourhoods ($46\%$ and $48\%$ respective improvement).

## Materials and methods

### Overview of Sarand

**Figure 1.**
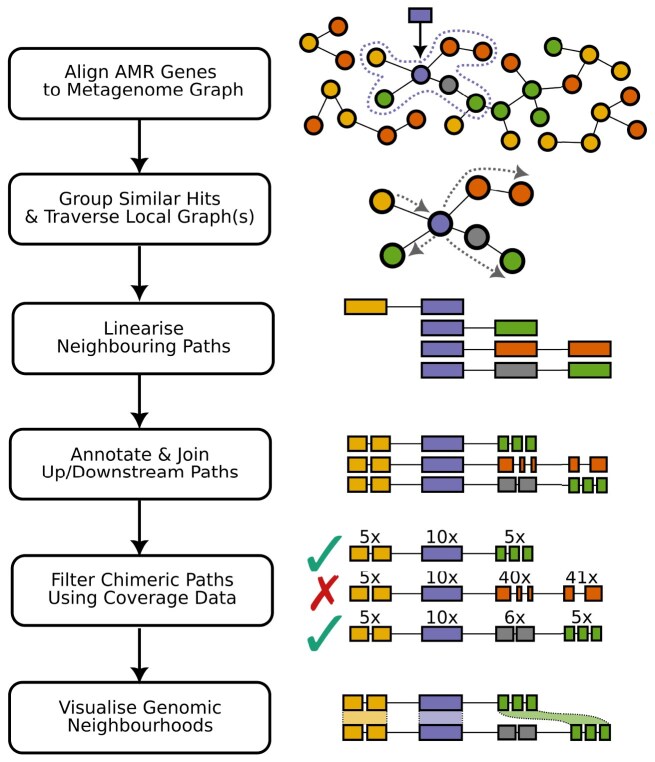
Overview of Sarand. Sarand ingests a GFA-formatted metagenome assembly graph with node coverage annotation. BLASTN-based [40] homology search of the graph is then performed using nucleotide ‘homolog model’ sequences from the CARD [41] and optionally either Bandage’s [42] graph-based BLAST implementation or GraphAligner [43]. Similar hits are grouped using sequence identity. The graph is then traversed for each group to extract the linear upstream and downstream neighbouring paths of the predicted AMR genes (within a user-specified sequence length). These upstream and downstream paths are annotated using Bakta [44] or Prokka [45] and (optionally) CARD’s RGI [41] before being paired into neighbourhoods. A differential gene coverage statistic is used to filter out improbable/misassembled neighbourhoods (i.e. potentially incorrectly matched upstream and downstream paths). Finally, extracted AMR neighbourhoods are visualized using the DNA-features-viewer library [46].

As shown in Fig. 1, Sarand starts with a coverage-annotated metagenomic assembly graph in GFA format generated by tools such as metaSPAdes [47], BCALM [38], or MEGAHIT [48]. In these assembly graphs, fragments of DNA sequences are presented as nodes (i.e. segments), while overlapping segments are connected via edges. A reference set of curated AMR genes is downloaded from the Comprehensive Antibiotic Resistance Database (CARD) [41] and aligned against the assembly graph using the graph-based BLASTN implementation in Bandage [42] (or optionally via an alternative GraphAligner query [43]). Then, for each predicted AMR gene, all paths of the graph within a given sequence length upstream and downstream of the nodes representing the AMR gene are traversed and extracted. These upstream and downstream paths are then annotated using either Bakta [44] or Prokka [45] and (optionally) the Resistance Gene Identifier (RGI) [49]. To generate genomic neighbourhoods, upstream and downstream paths are paired into valid neighbourhoods using a differential gene coverage statistic. Finally, the DNA-features-viewer [46] library is used to visualize the annotation of the extracted and filtered genomic neighbourhoods.

### AMR alignment and grouping

The Bandage implementation of BLASTN (or GraphAligner) returns a list of all possible paths in the graph aligning to reference AMR gene sequences with their corresponding sequence identity and query coverage. We retain all paths with identity and coverage above a user-supplied threshold (the default value for both parameters is 95%). Similar AMR sequences may align to the same graph nodes. To avoid redundancy, we group hits together that share the same paths at ${\gt}95\%$ identity and perform the neighbourhood sequence extraction task only once for each group.

### Extraction of gene neighbourhoods

#### Assembly graph processing

**Figure 2.**
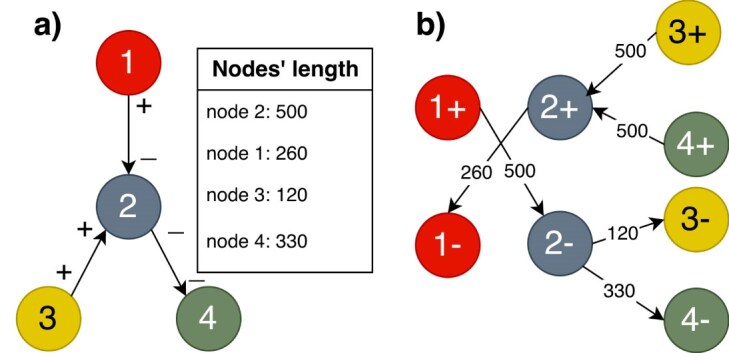
A toy example of conversion from (**a**) an assembly graph to (**b**) a weighted directed graph where the number of nodes and edges are doubled and the weight of each edge represents the length of the leading node.

GFA-formatted [50] assembly graphs consist of nodes (e.g. $A$ and $B$) representing individual nucleotide sequence segments and directed edges (e.g. $A \rightarrow B$), which indicate where the end of $A$’s sequence should be connected to the start of $B$’s sequence to form a path. However, as each node’s sequence can be represented in their original (indicated by ‘+’) or reverse complement (indicated by ‘−’) orientation, these edges are only defined in relation to specific node orientations (e.g. $A^+$ in positive orientation connecting to the reverse complement of $B^-$). In the GFA format this is annotated on the edge itself as a bi-directed graph [50, 51]. However, to simplify later traversals, we instead split each node into a pair of distinct nodes to separately represent both the original ($A^+$) and reverse complement orientation ($A^-$) and their associated simple directed edges (e.g. $A^+\rightarrow B^-$). Each node is annotated by the assembler with its estimated coverage but we also annotate (i.e. weight) each directed edge with the length of the target node’s sequence. For example, assuming the length of node $B$’s corresponding sequence is 250 nucleotides, the weight of the edge $A^+ \rightarrow B^-$ is set to 250. See Fig. 2 for a visual example of the graph processing we applied.

**Figure 3.**
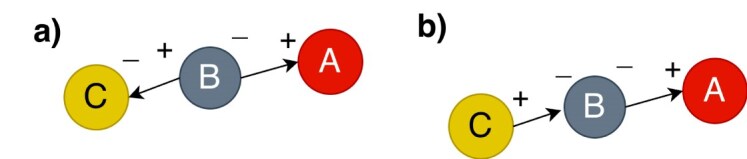
Utilizing reverse edges in the neighbourhood extraction process. In this example, segment $A$ contains an AMR gene, $B$ is a candidate upstream region of $A$, and $C$ is a candidate upstream region of $B$. The edge direction indicates how the nodes sequences are concatenated (for example, an edge from $B$ to $A$ means that the sequence of $B$ precedes that of $A$). The node orientation (sign) specifies the direction of each sequence; for instance, in $B^- \rightarrow A^+$, the extracted sequence is formed by concatenating the reverse complement of $B$’s sequence with $A$’s sequence, taking their overlap into account. (**a**) No path between $A$ and $C$ can be found by using the edges as they are available in the assembly graph, (**b**) by reversing the edge between $B$ and $C$ (i.e. taking the reverse complement of the edge’s sequence) and converting $B^+ \rightarrow C^-$ to $C^+ \rightarrow B^-$, we can make a path from $C$ to $A$.

#### Assembly graph traversal

To extract the neighbourhood sequences of an AMR gene in the processed graph, we use directed graph traversal algorithms based on node orientation. Specifically, two directed edges (e.g. $A \rightarrow B$ and $B \rightarrow C$) are connected in a path (e.g. $A \rightarrow B \rightarrow C$) only if the orientation of the common node (i.e. $B$) is the same in both edges. For example, with edges $A^+ \rightarrow B^-$ and $B^+ \rightarrow C^+$, there is no path between $A$ and $C$ through $B$, because the incoming and outgoing edges of $B$ correspond to conflicting orientations (‘−’ and ‘+’), breaking the continuity of the path. However, if an additional edge $B^- \rightarrow C^+$ exists in the assembly graph, then the path $A^+, B^-, C^+$ can be extracted. Additionally, a directed edge (e.g. $A^- \rightarrow B^+$) can be reversed to connect its constituent nodes in their opposite orientations (e.g. $B^- \rightarrow A^+$). The only difference is that the latter edge now represents the reverse complement sequence of the original edge.

Therefore, the main rules for extracting and extending upstream (or corresponding downstream) paths of a given node, $B$, are as follows:

- If there is an edge $C \rightarrow B$ where the orientation of $B$ in this edge is the same as its orientation in the part of the path that has already been extracted, $C$ is eligible to be considered in the upstream path. Similarly, if $B \rightarrow C$ then $C$ would be eligible as a downstream path.
- If there is an edge $B \rightarrow C$ (or $C \rightarrow B$) where the orientation of $B$ in this edge is the reverse of its orientation in the already extracted part of the path, $C$ with the reverse orientation is eligible to be considered in the upstream (or downstream) path.

As an example, let us assume that in Fig. 3, $A^+$ denotes the returned AMR gene path in the graph by the sequence alignment tool (i.e. Bandage-BLASTN or GraphAligner). To extract the upstream, we can include node $B^-$ because the orientation of $A$ in edge $B^- \rightarrow A^+$ is the same as the orientation of $A$ in the returned AMR gene path. However, as shown in Fig. 3a, it seems that no further nodes can be added to the upstream path as there is no edge from any other node to $B^-$. If we take advantage of the idea of reversing edges, then $B^+ \rightarrow C^-$ can be converted to $C^+ \rightarrow B^-$, as shown in Fig. 3b, and the sequence of $C^+$ can be added to the upstream path as well.

#### Neighbourhood extraction algorithm

Using the *ego_graph* function from the NetworkX [52] library in Python, we extract a subgraph centred on the AMR gene within a predefined path-weight threshold (i.e. the sum of edge weights in the path or the total neighbourhood nucleotide sequence length). From this subgraph, and using the AMR gene as the source node, we follow the rules defined above to traverse all paths with a total path weight less than or equal to the predefined neighbourhood sequence length are identified (default 1000 bp). To avoid redundant subpaths, we retain only the longest superpath amongst overlapping paths. For example, from the paths $[A^+, B^+, C^+, D^+]$, $[A^+, B^+, C^+]$, and $[A^+, B^+]$, only $[A^+, B^+, C^+, D^+]$ is kept.

Each graph path and its corresponding extracted sequence is added to a list of neighbourhood sequences. However, although these neighbourhoods are distinct at the graph level (i.e. non-redundant and containing non-identical segments), they can still result in highly similar nucleotide sequences when the differing segments are short. These differences may represent true biological variation such as strain-level diversity [53] but can also be due to sequencing and assembly error. Given the well-established challenge in distinguishing between these [31, 54], we took the common approach of collapsing this low-level variation (e.g. [47]). To do this we used CD-HIT [55] to cluster neighbourhood sequences at a user-defined sequence identity threshold, set to 90% by default, and selected the longest representative from each cluster for further analysis.

**Figure 4.**
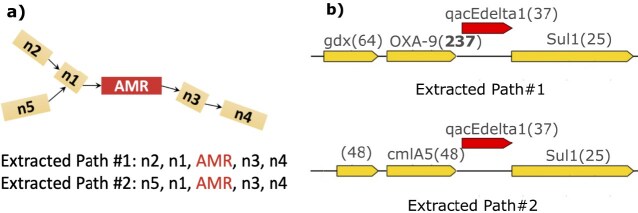
Neighbourhood extraction. (**a**) The neighbourhood of an AMR gene (*qacEdelta1*) is extracted as two separate paths with a user-specified maximum length (default 1000 bp) on each side of the AMR gene. (**b**) Two neighbourhood sequences are annotated with the numbers in parentheses presenting gene coverage. Since the difference in coverage depth for *OXA-9* (i.e. 237) and that of the *qacEdelta1* gene (i.e. 37) exceeds 30 (the default threshold value for Sarand), *OXA-9* and any gene in its upstream region are removed, with the corresponding upstream sequence removed from the candidate path list.

For each neighbourhood sequence, we extract the path, including the list of nodes, the sequence segments represented by each node, and the coverage (i.e. depth of coverage) for each node. Figure 4 illustrates an example of neighbourhood extraction. As some subgraphs can become highly complex, users can optionally set a time threshold (e.g. 10 min) for enumerating all upstream and downstream paths of a given AMR gene. If this threshold is exceeded, the search is terminated, and only the paths identified up to that point are returned.

### Comparing annotations

After extracting neighbourhood sequences for each AMR gene, we run Bakta or Prokka to identify gene information, including name, start and end positions in the sequence, length, and locus tag. Annotated protein sequences are then processed using CARD’s RGI to recognize any potential AMR gene; any strict/perfect annotations from RGI then supersede those identified by Bakta or Prokka. Although RGI also relies on alignment-based methods (e.g. BLAST/DIAMOND), it applies curated, gene-specific thresholds and captures additional resistance mechanisms (e.g. point mutations), providing a complementary and more standardized AMR annotation framework compared with Bandage-BLAST outputs alone. Only unique annotations are retained for different paths; by default, two annotations with the same number of genes are considered identical only if they share an identical annotation or have a pairwise BLASTN identity and coverage greater than 90%.

### Gene coverage and path filtering

In many cases, we expect the graph to contain false-positive chimeric paths (i.e. false pairings of upstream and downstream neighbourhoods, which do not exist within the underlying genomes) that require filtering. To reduce the number of such paths, we consider the relative abundance or *coverage* (i.e. depth of coverage) of each node, calculated as


1 \begin{eqnarray*}
\mathrm{coverage}_\mathrm{node} = \frac{\mathrm{kmerCount}_\mathrm{node}}{\mathrm{len(node)} - \mathrm{max(kmerSize)}},\end{eqnarray*}

where $\rm kmerCount_{node}$ is the number of times *k*-mers of the node appear in the reads, $\rm len(node)$ is the length of the node in nucleotides, and $\rm max(kmerSize)$ is the maximum *k*-mer size used by the assembler.

The coverage for each annotated gene is a weighted average over the coverage of all nodes covering the gene sequence, with the weight of each node expressed as the length of the gene sequence contained in the node divided by the total sequence length. For example, consider a gene of total length $L$, represented by the path $N_1 \rightarrow N_2 \rightarrow N_3$, where $L_1$, $L_2$, and $L_3$ nucleotides of the gene sequence are derived from nodes $N_1$, $N_2$, and $N_3$, respectively. In this case, the gene’s coverage is computed as


2 \begin{eqnarray*}
\mathrm{coverage}_\mathrm{gene} = \sum \limits _{i=1}^{3} \mathrm{coverage}_{N_i} \times \frac{L_i}{L} .
\end{eqnarray*}

We use a coverage difference threshold between the target AMR gene and other genes in each annotated extracted sequence to remove implausible paths. If the difference in coverage between gene $G$ and the target AMR gene exceeds this threshold, then gene $G$ and any other gene(s) in its upstream or downstream path are removed. This process has been illustrated with an example in Fig. 4b, where the *OXA-9* gene and all its upstream genes are removed from the extracted sequence due to the considerable difference in coverage between *OXA-9* and the target AMR gene (i.e. *qacEdelta1*). A default coverage difference of 30 was determined through calibration on simulated datasets (1_1_1 and CAMI_M_1 described below) to maximize precision and sensitivity (see Supplementary Figs S1 and S2). All remaining unique annotated sequences are returned by Sarand and visualized. We also compute an approximate estimate of the relative abundance of each neighbourhood by outputting their average coverage (defined as a length-weighted average of the node coverage values in the path).

### Validation of Sarand

We ran Sarand (v1.0.0) with GFApy v1.1.0 [56], Bandage v0.8.1 [42], Prokka v1.14.5 [45], RGI v5.1.1 [49], CARD v3.1.0 [41], and DNA_features_viewer v3.0.3 [46]. As presented in Table 1, we validated our software on three classes of datasets: (i) simple metagenomes simulated from either one or two strains from each of *Escherichia coli, Staphylococcus aureus*, and *Klebsiella pneumoniae* retrieved from RefSeq, designated as ‘1_1_1’ and ‘2_2_2’, respectively, with reads simulated using ART V2.5.8 [57] (HiSeq 2500 with read length = 150 bp, insert size = 500 bp, fold coverage = 20); (ii) ‘medium’- and ‘high’-complexity datasets from the Critical Assessment of Metagenome Interpretation (CAMI) study [58]; and (iii) three published metagenome samples [39] derived from urban sewage sequenced by Illumina HiSeq.

**Table 1.** Datasets simulated from RefSeq (1_1_1, 2_2_2), the CAMI Challenge [59], and real metagenomic samples selected from the Global Urban Sewage AMR Monitoring Project [39]

Name	Dataset composition	Annotated
		AMR genes
1_1_1	*E. coli* SMS-3-5 (NC_010498, NC_010488, NC_010485, NC_010486, NC_010487)*K. pneumoniae* MGH 78578 (NC_009648, NC_009649, NC_009650, NC_009651, NC_009652, NC_009653)*S. aureus* Mu50 (NC_002758, NC_002774)	378
2_2_2	1_1_1 plus *E. coli* UMN026 (NC_011751, NC_011749, NC_011739)*K. pneumoniae* HS11286 (NC_016845, NC_016838, NC_016846, NC_016839, NC_016840, NC_016847, NC_016841)*S. aureus* Mu3 (NC_009782)	451
CAMI_M_1	132 genomes	52
CAMI_M_2	132 genomes	54
CAMI_H_1	596 genomes	698
Urban	Albania (ERR1713331)	355
Sewage	Canada (ERR1713342)	101
	Cambodia (ERR1713370)	374

We examined the sensitivity and precision of Sarand on the simulated datasets (i) and (ii). To evaluate the validity of extracted sequences, we compared them with the ground truth available in the genomes from which these datasets were simulated. Precision measures the fraction of extracted upstream/downstream annotations that are valid (i.e. present in the underlying reference genomes), and sensitivity measures the fraction of upstream/downstream annotations from the reference that were identified by each method (i.e. Sarand, MetaCherchant, or contigs). For datasets 1_1_1 and 2_2_2, we used BLASTN v2.9.0 to query the AMR genes on the reference genomes with minimum coverage and sequence identity thresholds set to 95% and then extracted their neighbourhood sequences to be compared with Sarand’s extracted neighbourhoods. For the CAMI dataset, extracted neighbourhoods passing the Sarand thresholds were similarly compared against the gold-standard FASTA files available from the CAMI website. If an upstream (or downstream) sequence from the assembly graph matched true upstream (or downstream) sequences from the reference genomes, it was considered a true positive; extracted sequences with no match in the ground truth were counted as false positives.

We compared Sarand against contigs assembled by metaSPAdes v3.14.1 [47] with default parameters, as well as two recently developed tools representing different paradigms: MetaCherchant [34] and Spacegraphcats [37]. To extract neighbourhood sequences from contigs, we first ran the *makeblastdb* command on the contigs file to build a local BLAST database. We then used BLASTN v2.9.0 to query the AMR genes against the contigs. For each identified AMR gene, the corresponding neighbourhood sequences of a specified length were extracted from the same contig.

For MetaCherchant v0.1.0, we used the default settings ($k = 31$, $\mathrm{maxkmers} = 100000$, $\mathrm{bothdirs} = \mathrm{false}$, $\mathrm{chunklength} = 10$) with $\mathrm{coverage} = 1$ to include as many *k*-mers as possible in the results as recommended by the developers (personal communication). For a given AMR gene, MetaCherchant will produce a local neighbourhood graph but does not extract the actual neighbourhood sequences. Therefore, to have results comparable to contig and Sarand-based analyses, we extracted and annotated neighbourhoods from MetaCherchant-generated graphs using Sarand (further details can be found in Section D of the Supplementary Materials).

We ran Spacegraphcats v2.0.12 for datasets 1_1_1 and 2_2_2 with $\mathrm{radius}=10$ and $k=31$. For the more complex CAMI datasets, a $\mathrm{radius}$ greater than 1 exceeded our 1.4 TB available memory, so $\mathrm{radius}$ 1 was used instead. Similar to MetaCherchant, Spacegraphcats does not return linear sequences but the subgraph containing the query AMR gene and surrounding nodes up to the $\mathrm{radius}$. Therefore, we applied Sarand to these subgraphs to linearize, filter, and annotate the neighbourhood sequences (Supplementary Materials, Section D). However, in almost all cases, the length of the extracted neighbourhood sequence was not long enough to annotate any gene(s).

All experiments were run on a computer workstation running Ubuntu 20.04 with 3.5 GHz Intel Xeon quad-core processor and 128 GB RAM, except for CAMI experiments with Spacegraphcats that required a significant amount of memory and were run on Ubuntu 20.04 server with 88 Xeon Processor cores and 1.4 TB memory.

## Results

### Performance on simulated datasets

#### Identification of AMR genes

To establish a performance baseline and ground truth, we compared the sensitivity of BLASTN for putative AMR gene detection in the underlying reference genomes, metaSPAdes assembly graphs (using Bandage + BLASTN), and metaSPAdes contigs across the simulated datasets (Table 2). Although nearly all AMR genes identified in the underlying reference genomes were also detected in the assembly graphs and contigs, there was a general trend of decreasing numbers of detectable AMR genes (reference genome $\ge$ assembly graph $\ge$ contigs) across all datasets. This supports our hypothesis that some AMR sequences are lost in the process of extracting contigs from the assembly graph.

**Table 2.** AMR genes identified by BLASTN in each dataset

Dataset	Reference	Assembly graph	Contigs
1_1_1	378	378	376
2_2_2	451	451	445
CAMI_M_1	52	48	46
CAMI_M_2	54	45	44
CAMI_H_1	698	677	675

#### Extraction of AMR gene neighbourhoods

Using the underlying reference genomes as the ground truth, we then evaluated the performance of Sarand, MetaCherchant, and metaSPAdes contigs in correctly extracting 1000 bp upstream and downstream AMR gene neighbourhoods. We did not include the results from Spacegraphcats as almost all neighbourhood sequences extracted by Spacegraphcats were not long enough to contain any identifiable neighbourhood genes.

In this analysis, precision and sensitivity are computed at the neighbourhood annotation level rather than at the individual gene sequence level. Specifically, for each AMR gene, we compare the extracted upstream and downstream neighbourhood annotations to the corresponding upstream and downstream neighbourhood annotations from the same region in the underlying reference genome. A true positive corresponds to an extracted upstream (or downstream) neighbourhood annotation set that matches the reference neighbourhood annotations. A false negative corresponds to a reference neighbourhood that was not correctly reconstructed, and a false positive corresponds to an extracted neighbourhood that does not match any reference neighbourhood. Therefore, precision measures the fraction of extracted neighbourhood annotations that correctly match the reference, and sensitivity measures the fraction of reference neighbourhood annotations that were successfully reconstructed.

**Figure 5.**
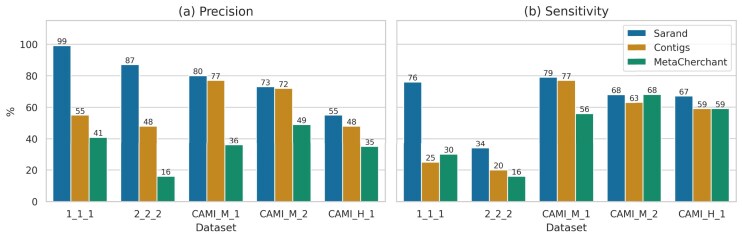
Performance comparison across simulated datasets. (**a**) Average precision. Precision measures the fraction of extracted upstream/downstream annotations from each method (i.e. Sarand, MetaCherchant, or contigs) that were present in the underlying reference genomes. (**b**) Average sensitivity. Sensitivity measures the fraction of upstream/downstream annotations from the reference that were identified.

Precision (Fig. 5a) and sensitivity (Fig. 5b) were then compared across these methods. Sarand outperformed MetaCherchant and the contig-based approach on the simplest datasets, with near-perfect precision and 76% sensitivity on the 1_1_1 dataset. Contig-based analyses were next best-performing with 55% precision and 25% sensitivity. Adding a second genome of each of the three species led to a drastic reduction in accuracy: while the precision of Sarand remained relatively high at 87%, the sensitivity dropped to 34%. The performance of both other methods uniformly decreased as well.

When applied to the CAMI datasets, Sarand remained the top-performing method in terms of precision and sensitivity, albeit with much smaller margins relative to the contig-based measures. Specifically, Sarand achieved 79% (68%) sensitivity across CAMI_M_1 (CAMI_M_2), compared with 77% (63%) for contig-based and 56% (68%) for MetaCherchant. Consistent with the results on the simple simulated datasets, the performance of all methods tended to decrease with increasing complexity (i.e. from CAMI_M_1 to CAMI_M_2 to CAMI_H_1), with the notable exception of MetaCherchant, which performed better on CAMI_M_2 than on CAMI_M_1 in terms of both precision and sensitivity.

We also compared the number of AMR genes whose upstream and downstream neighbourhoods were correctly extracted across each method and dataset. This was summarized across two different sensitivity thresholds: number of AMR genes with all their upstream and downstream neighbourhoods detected successfully, and number of AMR genes with at least 50% of their upstream or downstream neighbourhoods detected successfully (Supplementary Figs S3–S12). For 1_1_1 and 2_2_2, Sarand was able to fully reconstruct the neighbourhood of more AMR genes than contigs or MetaCherchant. It also identified at least $50\%$ of the true neighbourhoods for $86\%$ of AMR genes in 1_1_1 and $37\%$ of AMR genes in 2_2_2 that were detected by neither contigs nor MetaCherchant. In the CAMI datasets, MetaCherchant was able to reconstruct the most full neighbourhoods (2%, 11%, and 23% of AMR genes for CAMI_M_1, CAMI_M_2, and CAMI_H_1, respectively). However, as shown in Fig. 5b, when comparing across all simulated samples, Sarand identifies neighbourhoods with higher sensitivity than MetaCherchant and contigs.

To further disentangle the impact of graph-based neighbourhood extraction from downstream gene annotation, we additionally evaluated performance at the sequence level (Supplementary Section C; Supplementary Figs S13 and S14). In this analysis, we compared extracted neighbourhood sequences directly to the corresponding reference sequences, independent of gene annotation. Across most datasets, particularly 2_2_2, sequence-level sensitivity was substantially higher than annotation-level sensitivity. This indicates that in many cases the correct neighbourhood sequences were successfully reconstructed by Sarand, but some genes within those sequences were not detected during the annotation step. These results suggest that reduced sensitivity is largely attributable to annotation limitations rather than failures in graph traversal or neighbourhood extraction.

**Figure 6.**
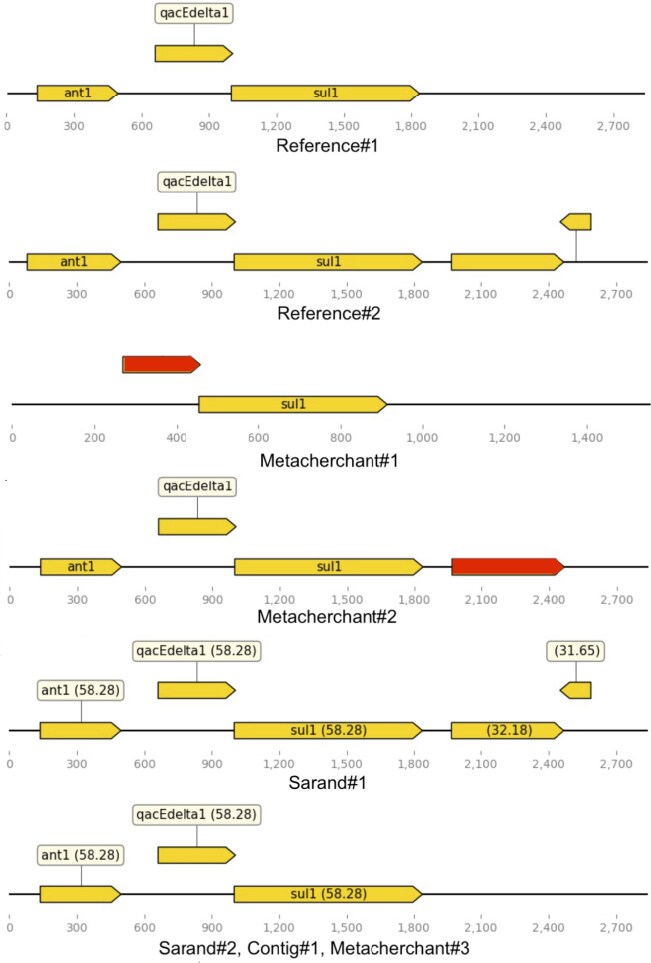
Sul1 neighbourhood comparison. The annotation of neighbourhood sequences extracted for *sul1* from CAMI_H_1 by Sarand, MetaCherchant, and contigs. The Sarand#2, Contig#1, and MetaCherchant#3 neighbourhoods include only the upstream sequence. False-positive genes predicted by MetaCherchant are highlighted in red.

**Figure 7.**
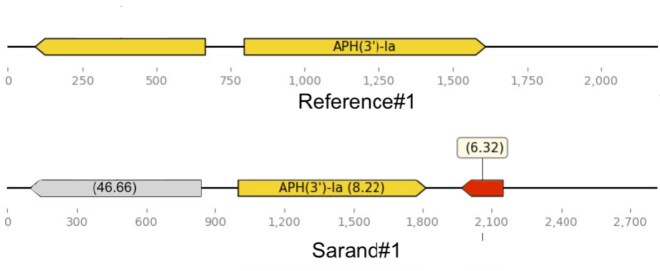
Sarand False Negative neighbourhood. The annotation of neighbourhood sequences extracted for *APH(3′)-Ia* from Sarand and a reference sequence from the CAMI_H_1 dataset. The gene highlighted in red is a false-positive case, while the gene highlighted in grey was initially identified by Sarand but removed after applying Sarand’s differential gene coverage statistic.

We then investigated and compared neighbourhoods generated for specific AMR genes across our 3 methods. For *sul1* in CAMI_H_1 (Fig. 6), Sarand successfully reconstructed both reference neighbourhoods, whereas contig-based analyses only recovered one of these neighbourhoods. MetaCherchant identified three neighbourhoods; however, two of these contained false-positive genes that were absent from the reference. Conversely, Fig. 7 illustrates a case where Sarand, contigs, and MetaCherchant all fail to identify the correct neighbourhood.

To ensure a fair comparison across methods, we explicitly report the runtime and memory consumption associated with MetaSPAdes, since both Sarand and the contig-based approach depend on an assembler to generate either the assembly graph or contigs prior to processing. In contrast, because MetaCherchant produces only subgraphs and does not extract neighbourhood sequences, its runtime consists of two components: the time required to run MetaCherchant itself and the additional time needed for Sarand to perform neighbourhood extraction and annotation on the MetaCherchant-generated graphs (with a 2-min time limit per AMR gene). For memory usage, we report only the peak memory consumed during the MetaCherchant run, as this step dominates the memory footprint and exceeds the memory required for Sarand’s subsequent neighbourhood extraction.

In terms of performance Sarand achieves a highly competitive, and often superior, runtime profile, outperforming MetaCherchant on most datasets, including challenging large-scale assemblies such as CAMI_H_1. The memory results are similarly favourable: although Sarand inherits the MetaSPAdes memory footprint required by all assembler-dependent methods, its overall memory usage remains comparable to that of MetaCherchant. Together, these results highlight Sarand’s efficiency, scalability, and practicality for comprehensive neighbourhood sequence extraction in both large and resource-constrained metagenomic settings. More details can be found in Section E of the Supplementary Materials (Supplementary Figs S15 and S16).

### Detection of AMR gene neighbourhoods in sewage samples

We applied Sarand to three published metagenome samples from urban sewage sequenced using the Illumina HiSeq [39]. We chose three published samples from disparate geographic locations with different numbers and types of predicted AMR genes: sample ERR1713331 from Albania (355 AMR genes), sample ERR1713342 from Canada (101 AMR genes), and sample ERR1713370 from Cambodia (374 AMR genes). After assembly in metaSPAdes, we ran Sarand for the constructed assembly graph of each sample and extracted the list of filtered neighbourhood sequences of each AMR gene, based on a gene-coverage difference threshold of 30 (see the ‘Materials and methods’ section). Given the complexity of these graphs, Sarand was run with time limit of 10 min to extract upstream and downstream neighbourhood sequences for each AMR gene.

#### Presence of extracted sequences in reference databases

Although we anticipate that previously undiscovered AMR gene neighbourhoods will be present in complex, global metagenome samples, we can still assess the performance of Sarand in recovering neighbourhoods that are already present in reference databases. To assess this, we searched the neighbourhoods extracted by Sarand against the NCBI ‘nt’ database (August 2021) using BLASTN with a percent-identity threshold of 90%. We classified hits as ‘full match’ if the entire predicted neighbourhood of a given AMR gene was present in the database, ‘upstream + AMR’ or ‘AMR + downstream’ if only the upstream or downstream neighbourhoods predicted by Sarand, along with the AMR sequence, were found in the database, or ‘no match’ if neither the upstream nor downstream predicted neighbourhoods were present.

**Figure 8.**
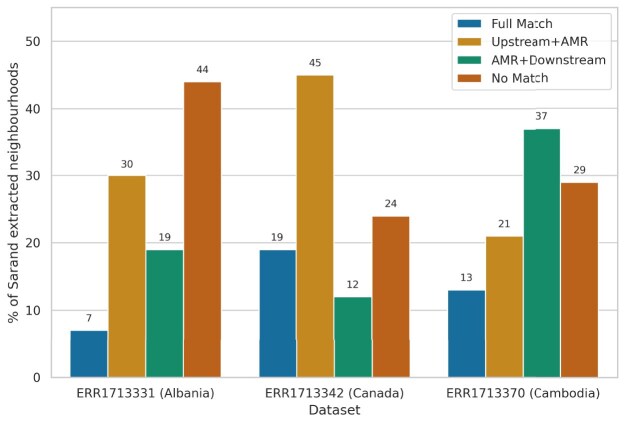
Sarand neighbourhood extraction from urban sewage. Summary results of BLAST hits for all neighbourhood sequences extracted from sewage samples. BLASTN was used to search the neighbourhoods extracted by Sarand against NCBI ‘nt’ database and the results were classified as ‘full match’ (the entire sequence is present in the database), ‘upstream + AMR’ (only upstream sequence is present), ‘downstream + AMR’ (only downstream sequence is present) and ‘no hit’ (neither upstream nor downstream is present).

The summary of the best BLAST hit results across all extracted neighbourhood sequences for every sample is shown in Fig. 8. In general, for sample *ERR1713331*, $56\%$ of the extracted neighbourhood sequences have at least partial matches, with more than half of ‘no match’ sequences belonging to the OXA beta-lactamase family. In samples *ERR1713342* and *ERR1713370* about three quarters of the extracted sequences had at least partial (i.e. upstream + AMR or AMR + downstream) matches, although full matches were fewer in number than either upstream or downstream matches.

## Discussion

This study, in line with other related work [34, 37, 60], showed that deriving gene neighbourhoods directly from the assembly graph can be more informative than exclusively relying on contigs. Comparing AMR neighbourhoods across all five different simulated datasets used in this study, Sarand performs on average 47% and 46% better than assembled metagenomic contigs alone in terms of precision and sensitivity, respectively. Similarly, given the 134% precision and 48% sensitivity improvement over MetaCherchant, we showed that methods making use of the full metagenomic assembly graph are better able to capture gene neighbourhoods than local assemblies. Unfortunately, we were not able to fully evaluate Spacegraphcats because for larger datasets, such as CAMI samples, it was too memory-intensive to run with comparable neighbourhood sizes (i.e. with radius${\gt} 1$). It may be possible to improve the performance of their dominating set approach for this specific problem. This could involve more aggressive denoising of the assembly graph and additional heuristics more optimized to individual gene-scale neighbourhoods rather than the genome-scale queries for which Spacegraphcats was developed. Similarly, we note that Sarand’s memory usage on large datasets (e.g. CAMI) can be substantial; however, this largely reflects the inherent computational requirements of working with full metagenomic assembly graphs and their associated analysis pipelines. As shown in Supplementary Fig. S16, the memory required to construct these graphs (via metaSPAdes) for high-complexity datasets is up to $100\times$ higher than the memory directly used by Sarand for neighbourhood extraction.

To address the performance of Sarand on real metagenomic data, we examined three wastewater metagenomic samples. 56%–76% of Sarand neighbourhoods were at least partially identifiable in NCBI deposited genomes, supporting Sarand’s ability to detect true neighbourhoods in environmental samples. Although it is still an open research question to robustly define a biologically relevant AMR gene neighbourhood, these results show that even partial metagenomic neighbourhoods can often be directly related to their wider-scale genomic context using reference databases. Future work could use this to further refine the delineation of AMR gene neighbourhoods, extend extracted neighbourhoods, and incorporate sequenced mock microbial communities (e.g. ZymoBIOMICS Microbial Community Standards). We also only evaluated one homology-search paradigm (i.e. Bandage’s BLAST implementation) and one input metagenomic graph (i.e. MetaSPAdes) despite Sarand supporting GraphAligner and MEGAHIT or BCALM graphs. A formal evaluation and comparison of the many emerging graph homology search methods (e.g. Wheeler graph-based methods [61], hidden Markov models [62], and *k*-mer methods [63]) would be a valuable resource to tool developers. Finally, while the current study did not assess Sarand’s adaptability to long-read metagenomic assembly graphs, this represents a promising direction for future work as long-read sequencing becomes increasingly prevalent.

## Conclusion

In conclusion, Sarand combines the sensitivity of read-based approaches with the genomic context provided as assemblies to profile AMR genes and their associated gene neighbourhoods from metagenomic samples. Across several simulated datasets, we were able to show that Sarand had a higher precision and sensitivity than alternative methods (e.g. MetaCherchant, metaSPAdes assembly, and Spacegraphcats). Future work will seek to extend the coverage threshold approach to further reduce chimeric neighbourhoods (false joining of upstream and downstream neighbourhoods), incorporate alternative homology search algorithms, and support long-read datasets.

## Supplementary Material

lqag066_Supplemental_Files

## Data Availability

All underlying data used in this study are available via the INSDC accessions or CAMI dataset identifiers described in Table 1. The assembly graphs used for the experiments are available via OSF: https://osf.io/x3zf5. Source code is available at https://github.com/beiko-lab/sarand and https://doi.org/10.5281/zenodo.20394761.
