# Supplementary material for: Sarand: exploring antimicrobial resistance gene neighbourhoods in complex metagenomic assembly graphs
Source: NAR Genom Bioinform. 2026 Jul 14;8(3):lqag066. doi: 10.1093/nargab/lqag066 (PMC13366076; doi:10.1093/nargab/lqag066)
Supplement: lqag066_Supplemental_Files [file lqag066_supplemental_files.zip › Sarand_revised_supplementary_2.pdf]

Supplementary Material for:

Sarand: Exploring Antimicrobial Resistance Gene Neighbourhoods in Complex Metagenomic Assembly Graphs

Somayeh Kafaie[1,2], Shahlla Naseri[1,2], David Mahoney[2,3], Travis Gagie[2], Robert G. Beiko[2], and Finlay Maguire[2,3,*]

[1]Department of Mathematics and Computer Science, Saint Mary's University, Halifax, Nova Scotia, Canada

[2]Faculty of Computer Science, Dalhousie University, 6050 University Avenue, Halifax, Nova Scotia B3H 4R2, Canada

[3]Department of Community Health and Epidemiology, Dalhousie University, 6050 University Avenue, Halifax, Nova Scotia B3H 4R2, Canada

A - Choosing Gene Coverage Threshold

To determine the coverage ratio threshold for comparing the target AMR gene with other genes in each annotated extracted sequence and to eliminate implausible paths, we calculated the precision and sensitivity of the extracted neighbourhoods for samples 1_1_1 and CAMI_M_1 across different coverage ratio values. Precision and sensitivity were selected as evaluation metrics since they are the primary performance measures used throughout the manuscript to assess Sarand's performance.

As shown in Figures S1 and S2, for two different datasets, 1_1_1 (a simulated dataset generated by us) and CAMI_M_1, the same threshold value performed consistently well, suggesting that the selected threshold is not highly dependent on the nature of the dataset. Based on these results, we selected a relative gene coverage threshold of 30 for Sarand to maximize precision while choosing a near-optimal value for sensitivity. Importantly, this threshold is not fixed. Users can adjust it using the `-c` or `--coverage_difference` parameter when running Sarand and evaluate performance across different threshold values depending on their specific dataset and analysis goals.

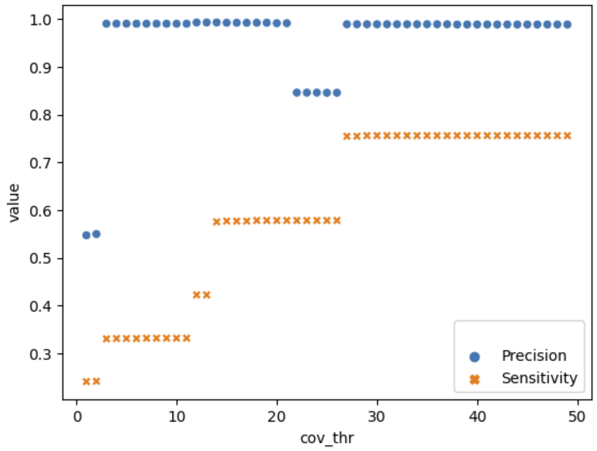

Figure S1: Precision and sensitivity of sample 1_1_1 for extracted neighbourhood sequences across different relative gene-coverage threshold values.

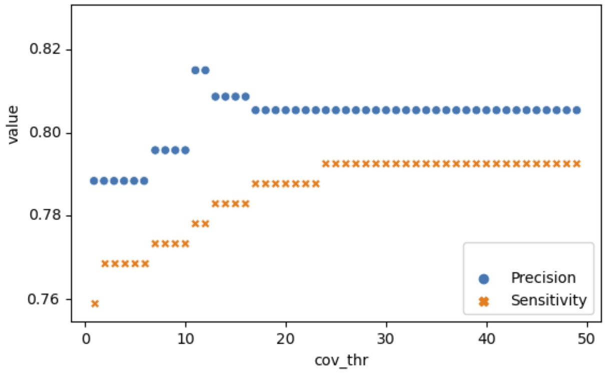

Figure S2: Precision and sensitivity of sample CAMI_M_1 for extracted neighbourhood sequences across different relative gene-coverage threshold values.

B - Comparison of Correctly Detected AMR neighbourhoods for Different Datasets

We compared the number of AMR genes with correctly extracted upstream and downstream neighbourhoods across methods and datasets, evaluated at two sensitivity thresholds: full detection and at least 50% detection of neighbourhoods. The results are summarized in Figures S3-S12.

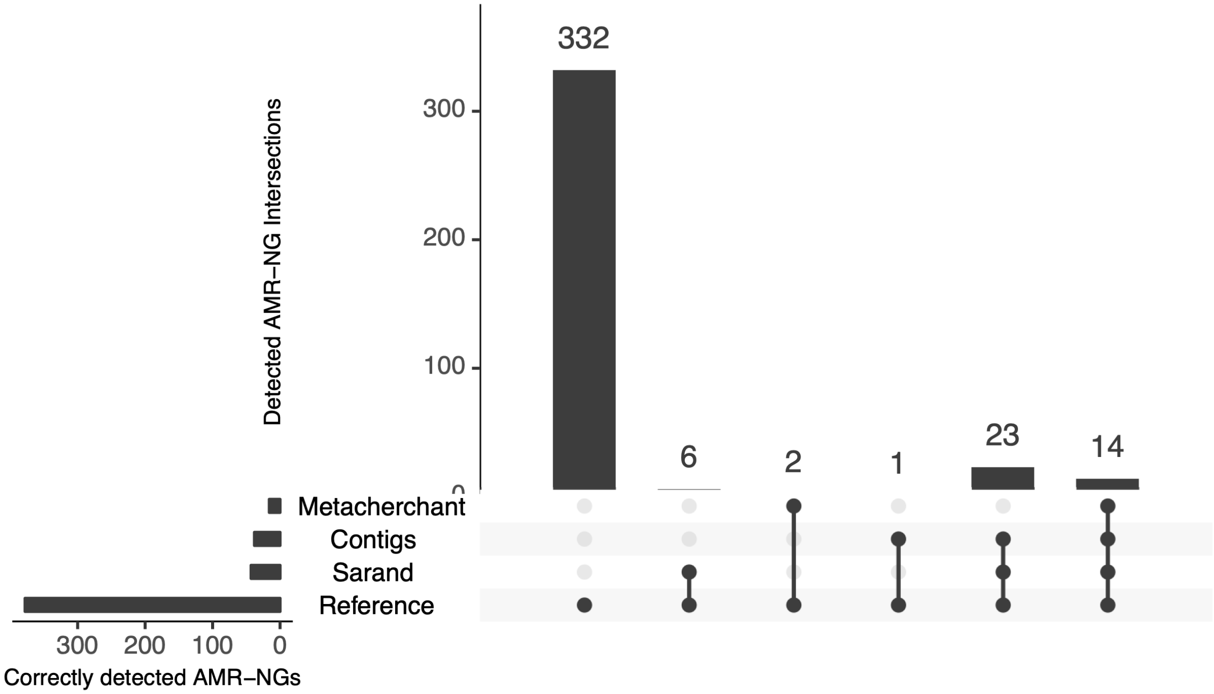

Figure S3: Comparison of correctly detected AMR neighbourhoods with sensitivity = 1 (i.e., all upstream and downstream neighbourhoods of the AMR gene are detected successfully) for 1_1_1 dataset.

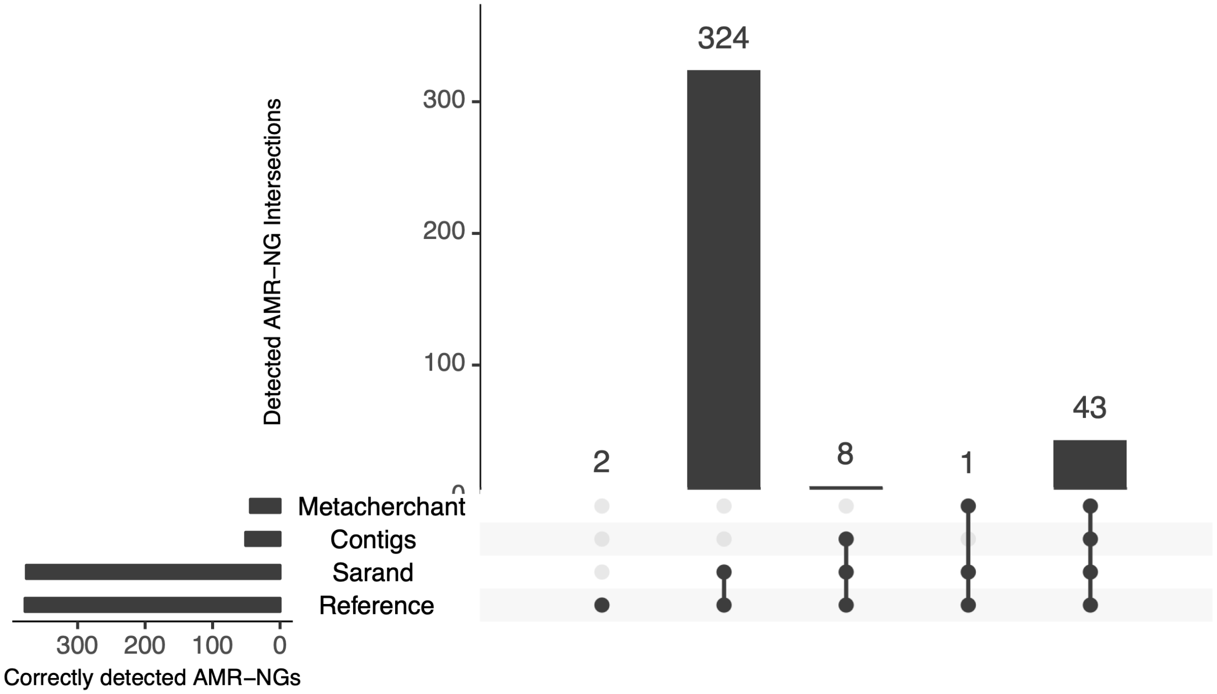

Figure S4: Comparison of correctly detected AMR neighbourhoods with sensitivity >= 0.5 (i.e., at least half of the upstream and downstream neighbourhoods of the AMR gene are detected successfully) for 1_1_1 dataset.

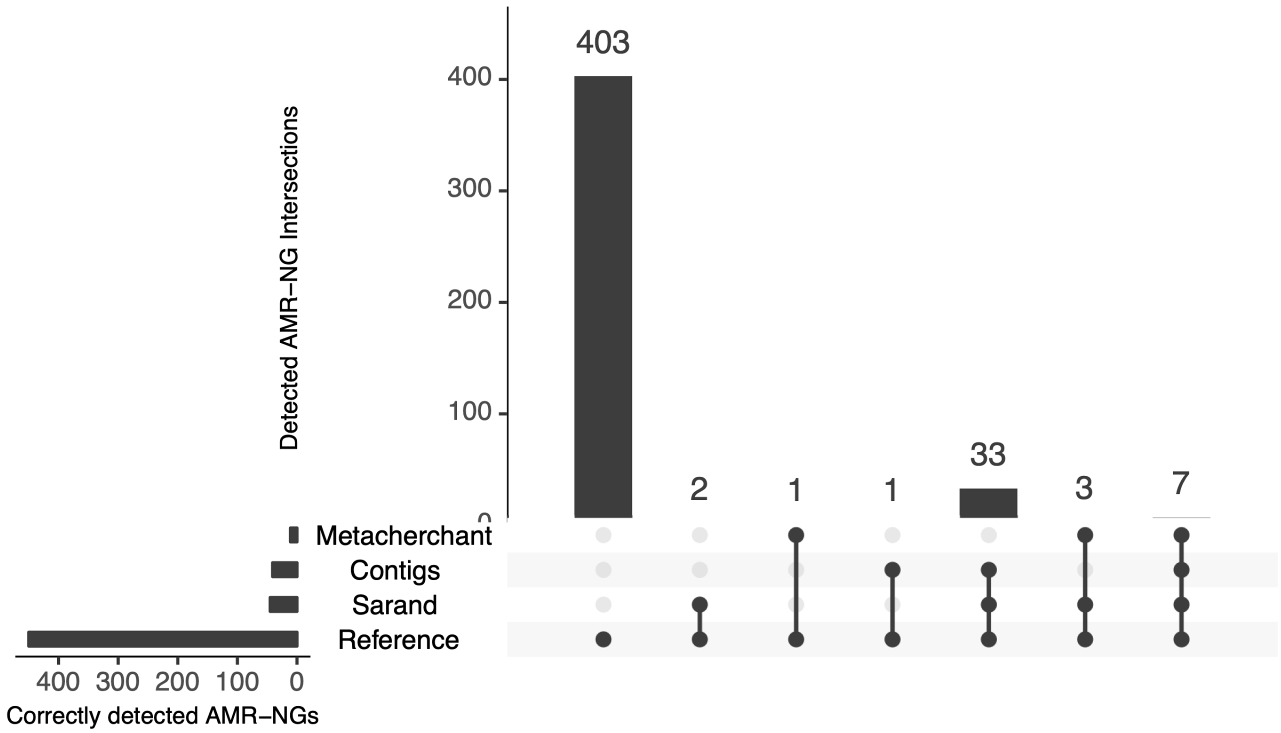

Figure S5: Comparison of correctly detected AMR neighbourhoods with sensitivity = 1 (i.e., all upstream and downstream neighbourhoods of the AMR gene are detected successfully) for 2_2_2 dataset.

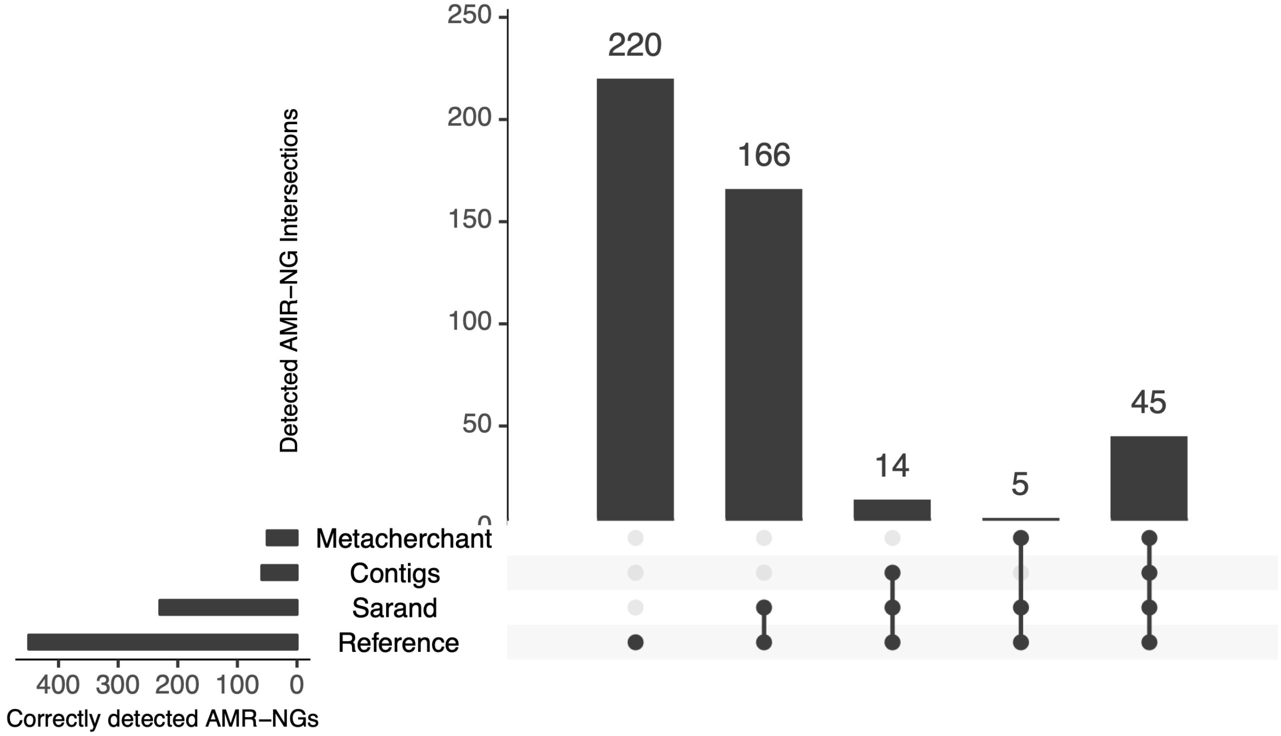

Figure S6: Comparison of correctly detected AMR neighbourhoods with sensitivity >= 0.5 (i.e., at least half of the upstream and downstream neighbourhoods of the AMR gene are detected successfully) for 2_2_2 dataset.

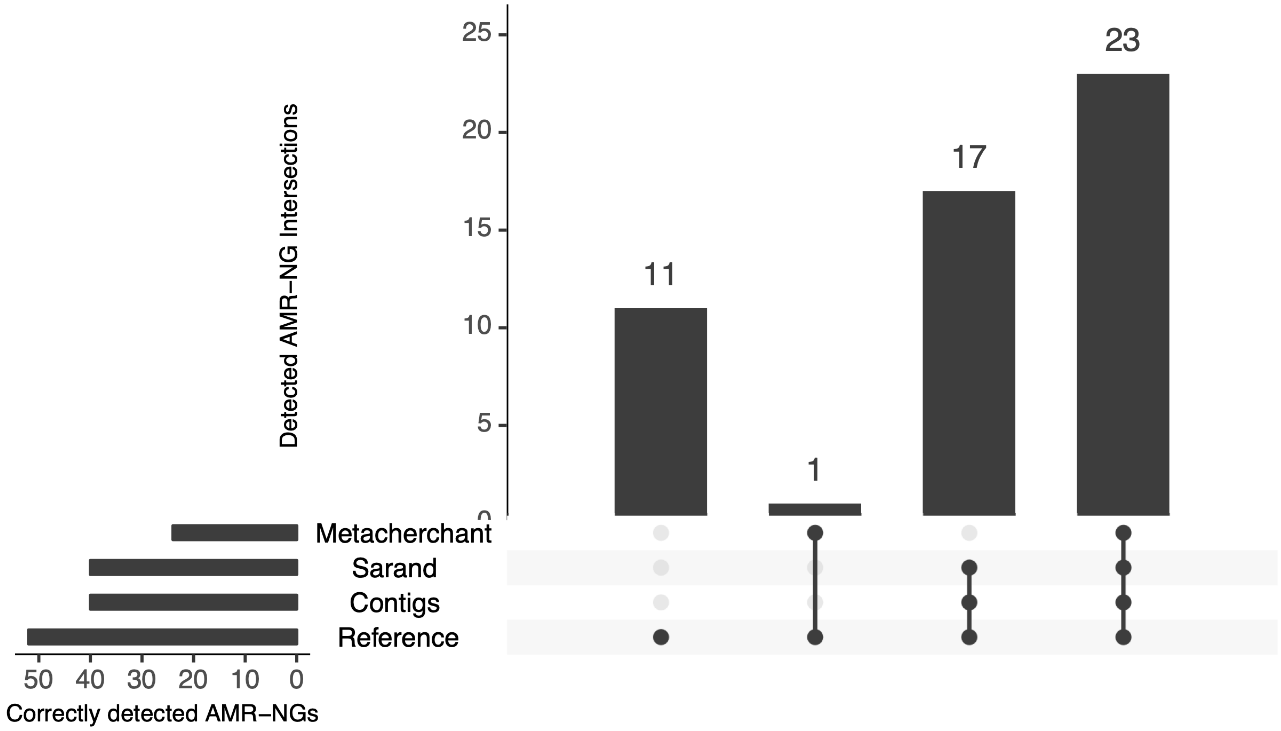

Figure S7: Comparison of correctly detected AMR neighbourhoods with sensitivity = 1 (i.e., all upstream and downstream neighbourhoods of the AMR gene are detected successfully) for CAMI_M_1 dataset.

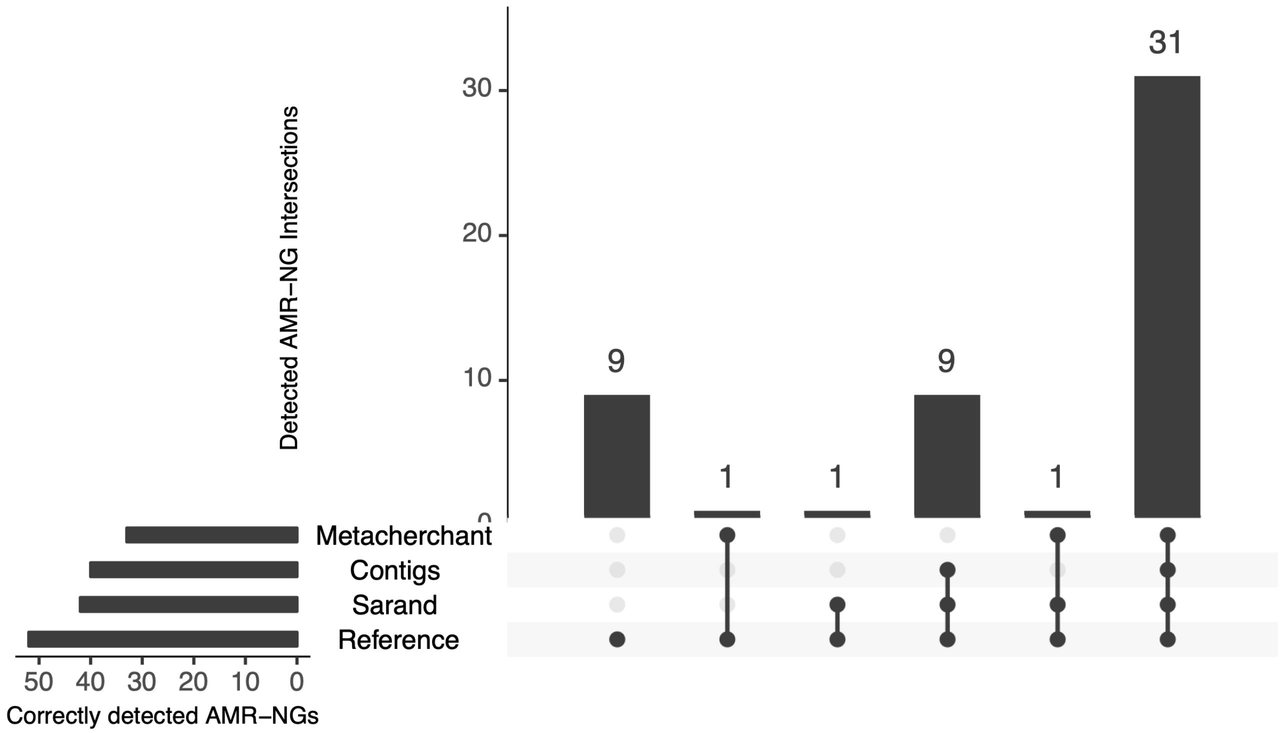

Figure S8: Comparison of correctly detected AMR neighbourhoods with sensitivity >= 0.5 (i.e., at least half of the upstream and downstream neighbourhoods of the AMR gene are detected successfully) for CAMI_M_1 dataset.

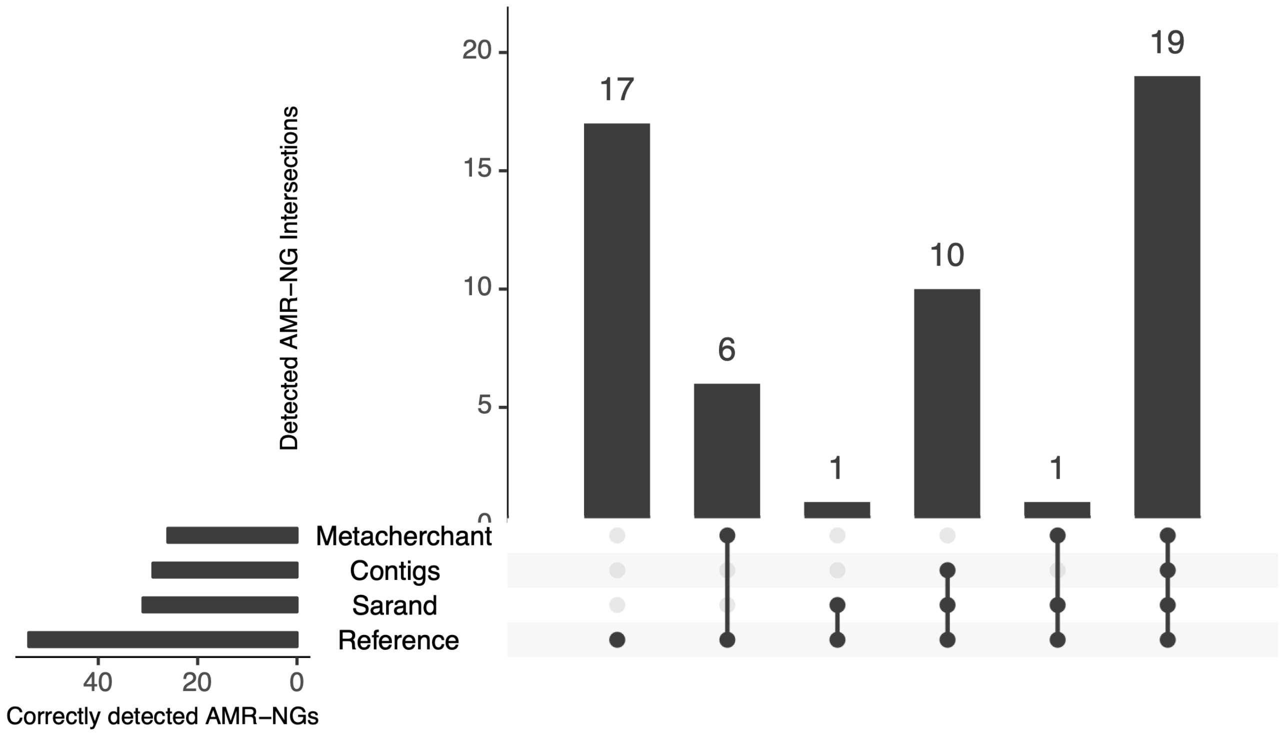

Figure S9: Comparison of correctly detected AMR neighbourhoods with sensitivity = 1 (i.e., all upstream and downstream neighbourhoods of the AMR gene are detected successfully) for CAMI_M_2 dataset.

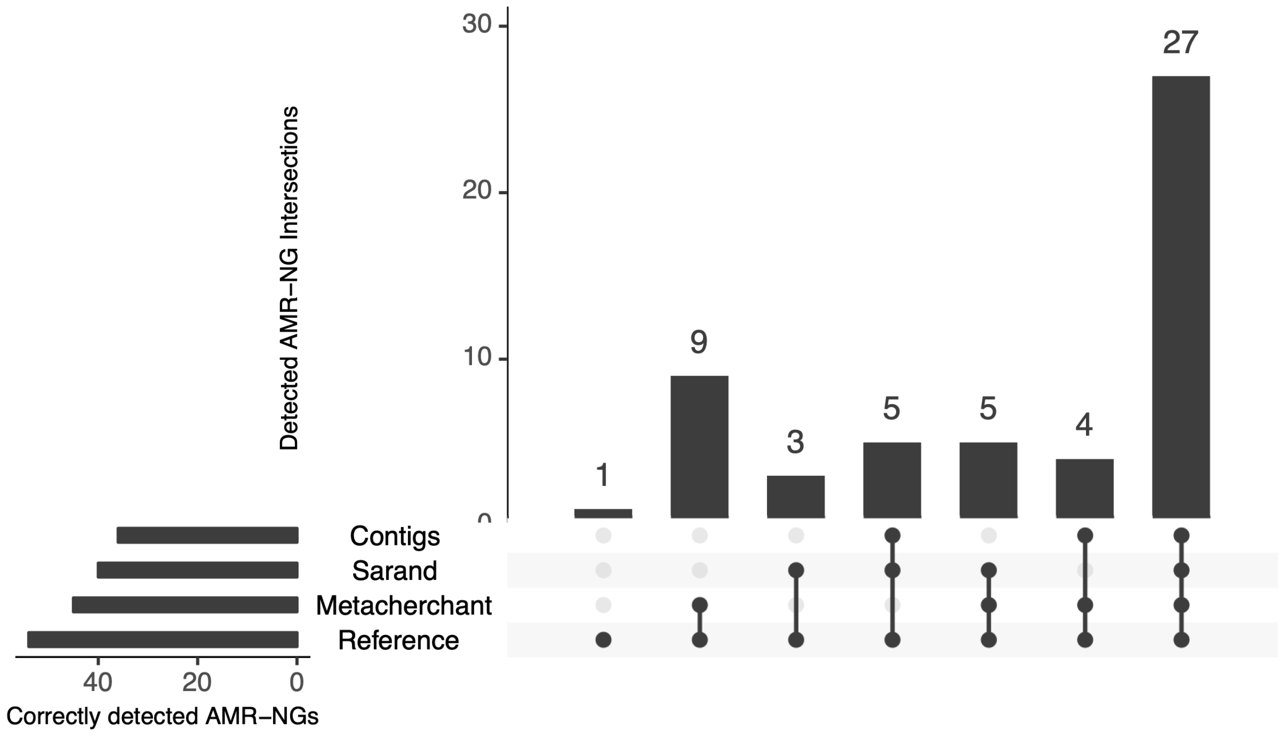

Figure S10: Comparison of correctly detected AMR neighbourhoods with sensitivity >= 0.5 (i.e., at least half of the upstream and downstream neighbourhoods of the AMR gene are detected successfully) for CAMI_M_2 dataset.

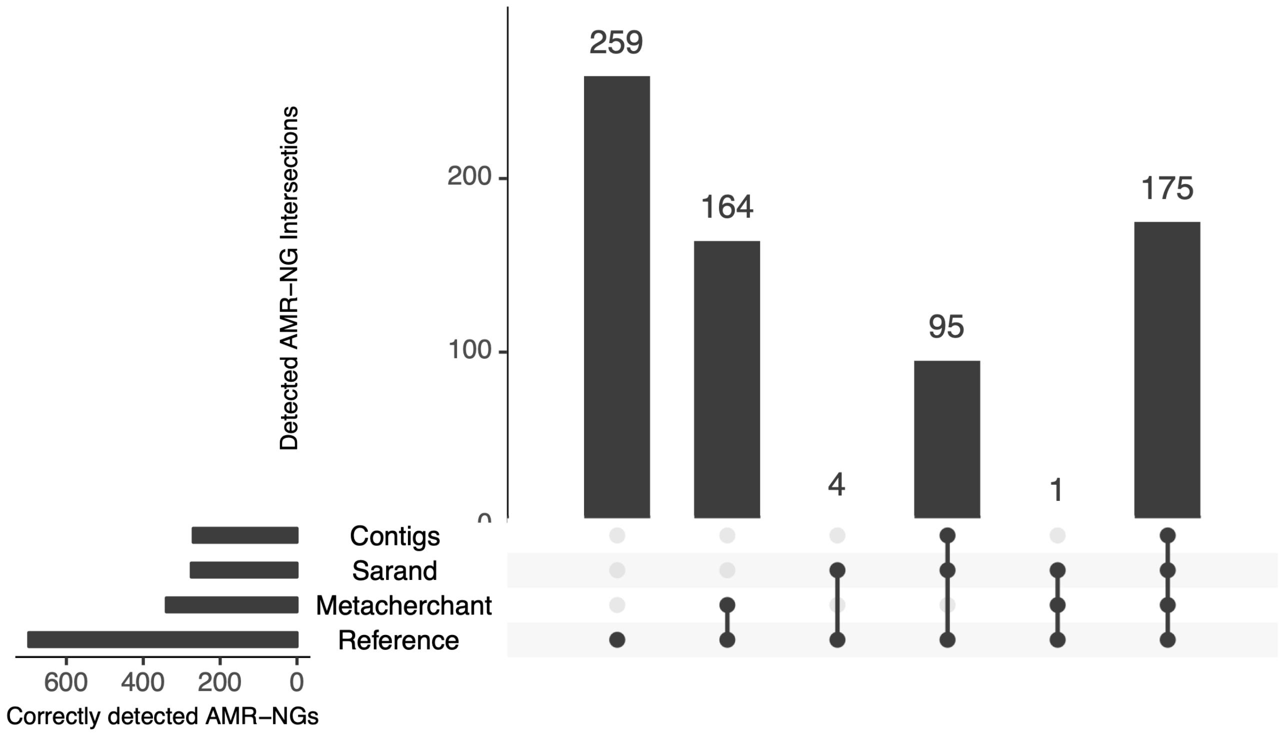

Figure S11: Comparison of correctly detected AMR neighbourhoods with sensitivity = 1 (i.e., all upstream and downstream neighbourhoods of the AMR gene are detected successfully) for CAMI_H_1 dataset.

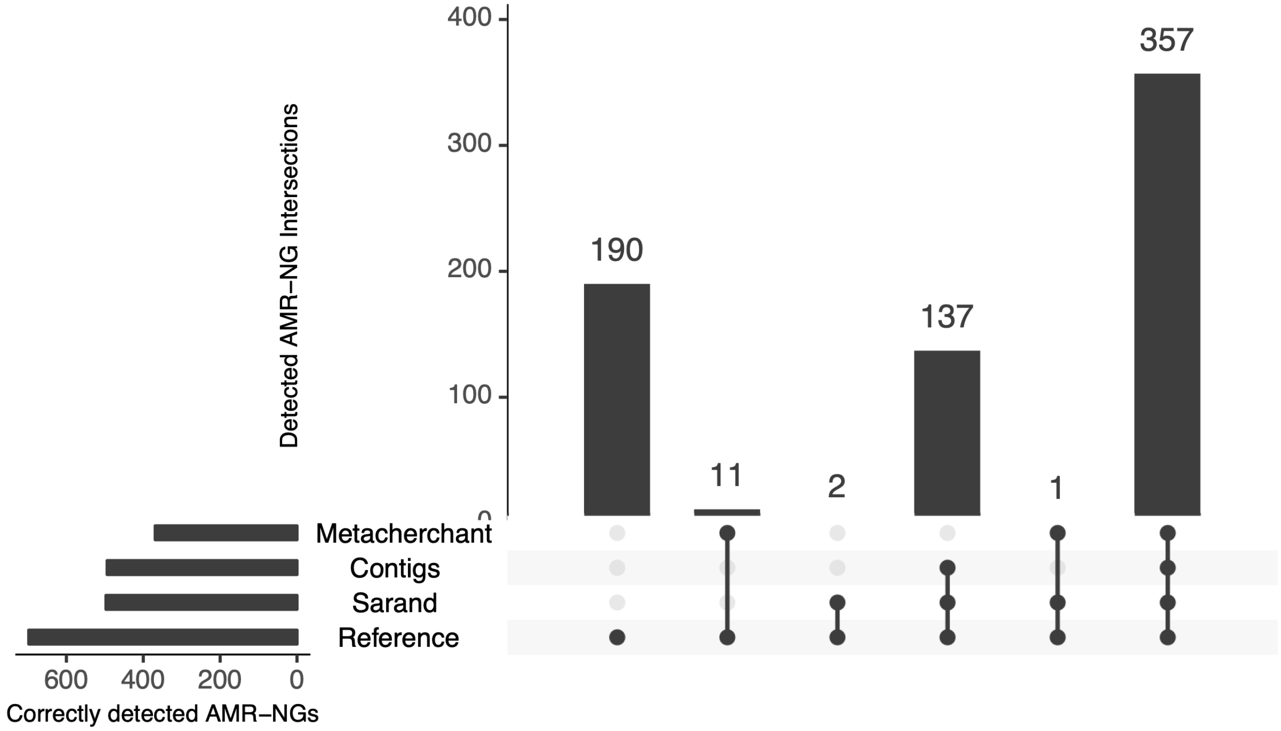

Figure S12: Comparison of correctly detected AMR neighbourhoods with sensitivity >= 0.5 (i.e., at least half of the upstream and downstream neighbourhoods of the AMR gene are detected successfully) for CAMI_H_1 dataset.

C - Sequence Comparison vs Annotation Comparison.

As explained in Materials and Methods, to validate Sarand, its extracted neighbourhood sequences are compared against those of the reference genomes. However, the comparisons are based on matching Prokka gene annotations between underlying reference and extracted neighbourhoods. To explore whether annotation inconsistencies were leading to neighbourhoods being incorrectly labelled as false, we also directly compared the upstream/downstream neighbourhood sequences. The sequence comparison was performed using BLASTN v2.9.0 and the threshold for identity and coverage was set to 90%. The comparison of these two cases for all simulated datasets in terms of precision and sensitivity are available in Figure S13 and Figure S14, respectively.

On the simple datasets 1_1_1 and 2_2_2, many more positive predictions were obtained by using sequence than when mapping annotations; the sensitivity on both datasets went from 76% and 34% to 90% respectively, while precision in both cases dropped below 50%. The difference on the CAMI_M datasets was never greater than 2%. The sequence-based approach yielded substantially higher precision (55% vs 92%) and sensitivity (67% vs 86%) on the CAMI_H_1 dataset. As an example, for a large group of SHV genes available in CAMI_H_1, Sarand recovered the sequence of the single neighbourhood present in the reference fully with identity and coverage equal to 100 (i.e., precision = sensitivity = 1 for sequence comparison). Comparing annotations, no annotation was found for the upstream sequences extracted from the reference and Sarand (i.e., no neighbourhood gene was found in the upstream sequence). However, regarding downstream sequences, given that Sarand's sequence is longer than the reference sequence, it includes one extra gene which is not present in the reference annotation and makes Sarand's annotation invalid (i.e., precision = sensitivity = 0 for annotation comparison). Supplementary CSV file "Supplementary Data: sup_data_annotation_vs_sequence.csv" shows different groups of AMR genes and Sarand's performance when comparing annotations vs sequences.

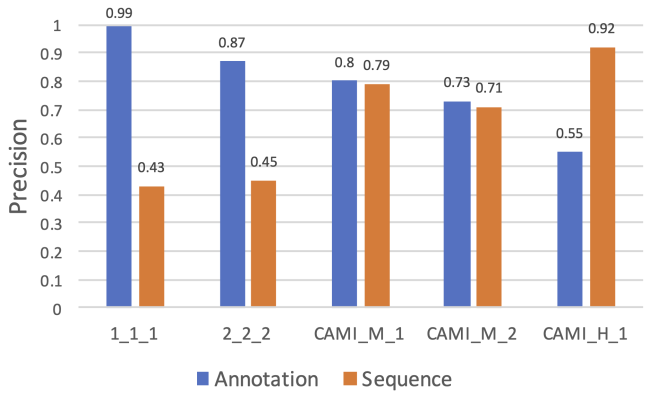

Figure S13: Precision of Sarand based on annotation vs sequence comparisons. For annotation evaluation, the gene-coverage threshold was set to 30, and for sequence evaluation the threshold for identity and coverage was set to 90%.

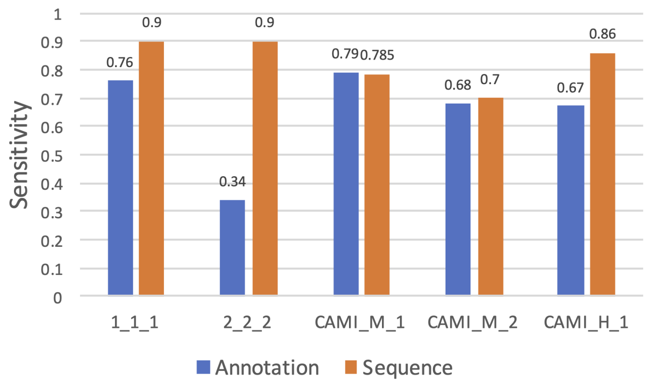

Figure S14: Sensitivity of Sarand based on annotation vs sequence comparisons. For annotation evaluation, the gene-coverage threshold was set to 30, and for sequence evaluation the threshold for identity and coverage was set to 90%.

D - Neighbourhood Extraction by Baselines

4.1 neighbourhood extraction from contigs

Using the `makeblastdb` command, we created a database from the list of contigs generated by MetaSPAdes for the given assembly graph. Next, the `blastn` command was used to align the query sequences (AMR genes) to the contigs, with identity and coverage thresholds set to 95%. Once aligned, the upstream and downstream neighbourhood sequences were extracted from the corresponding contigs, and nearly identical sequences were grouped using the same threshold as applied in Sarand. Finally, the extracted sequences were annotated and visualized.

4.2 neighbourhood extraction by MetaCherchant

For MetaCherchant v0.1.0, we used the default settings ($k = 31$, maxkmers = 100000, bothdirs = false, chunklength = 10) with coverage = 1 to include as many k-mers as possible, as recommended by the developers (*pers. comm.*). The following command was used:

```
./metacherchant.sh --tool environment-finder --k 31 --coverage=1  --reads
<readFile-path> --seq <geneSequenceFile-path> --output <outputDir>
--work-dir <workDir> --maxkmers=100000 --bothdirs=False --chunklength=10
```

For the CAMI databases, the default memory allocation was insufficient. To address this issue, we increased the memory allocation to 55.88 GB ($-m$ 60000000000) for CAMI_M_1 and CAMI_M_2. Similarly, for CAMI_H_1 we set the memory to 93.13 GB ($-m$ 100000000000). We then ran Sarand to extract all available neighbourhood sequences of a given AMR gene from the corresponding MetaCherchant graph.

This process used the constructed neighbourhood graph from MetaCherchant as input and aligned the AMR sequence to the local graph using the BLAST implementation in Bandage. In most cases, no path was returned, either because the number of nodes representing the AMR sequence in the MetaCherchant graph exceeded 50 (the maximum path length for Bandage+BLAST queries) or because no continuous path of nodes representing the AMR sequence existed in the graph.

Although MetaCherchant provides a list of nodes representing the AMR gene in their local graph, determining the order of these nodes is necessary to identify the first and last nodes in the path and extract the upstream and downstream sequences. To address this, we determined the order of the nodes by aligning their sequences to the AMR sequence. Then we ran our neighbourhood extraction functions to retrieve the upstream and downstream sequences. In most cases the local graphs produced by MetaCherchant are highly fragmented therefore the neighbourhood extraction process is time-consuming. We set a time limit of 2 minutes and extracted the upstream and downstream neighbourhood sequences for each AMR gene within that time frame. After filtering nearly identical sequences, the neighbourhood sequences were annotated and visualized.

4.3 neighbourhood extraction by Spacegraphcats

We ran Spacegraphcats v2.0.12 for datasets 1_1_1 and 2_2_2 with a radius of 10 and $k = 31$. However, for the CAMI datasets, we exceeded our 1.4TB memory limit when using a radius greater than 1. As a result, for the CAMI datasets, we were only able to run Spacegraphcats with a radius of 1.

For each dataset, Spacegraphcats first constructs a de Bruijn graph by running BCALM. After processing this graph, it outputs a list of nodes containing the neighbourhood of each query (AMR sequence). We extracted a subgraph from the original BCALM graph, consisting of the output nodes and their one-hop neighbors.

Sarand was then applied to extract the neighbourhood sequences of the target AMR gene from the subgraph. This process involved identifying the target AMR gene in the subgraph and running our neighbourhood extraction method to retrieve its upstream and downstream sequences up to the specified length. Finally, the extracted sequences were filtered (grouping nearly identical sequences), annotated, and visualized. However, in almost all cases, the length of the extracted neighbourhood sequence was not long enough to be annotated as any gene(s).

E - The Comparison of Runtime and Memory Consumption Across all Datasets

As shown in Figures S15 and S16, since both Sarand and the contig-based method rely on an assembler to generate the assembly graph or contigs, the runtime and memory consumption of MetaSPAdes is reported as well. Furthermore, because MetaCherchant only produces subgraphs and does not extract neighbourhoods, we have reported both the time required to run MetaCherchant itself and the time needed to run Sarand on the MetaCherchant-generated graphs to actually extract and annotate neighbourhood sequences (with a 2-minute time limit per AMR gene). Regarding memory consumption, because executing MetaCherchant itself requires substantially more memory than the subsequent neighbourhood extraction performed by Sarand on its output graphs, we only report the memory usage of running MetaCherchant (i.e., the peak memory usage).

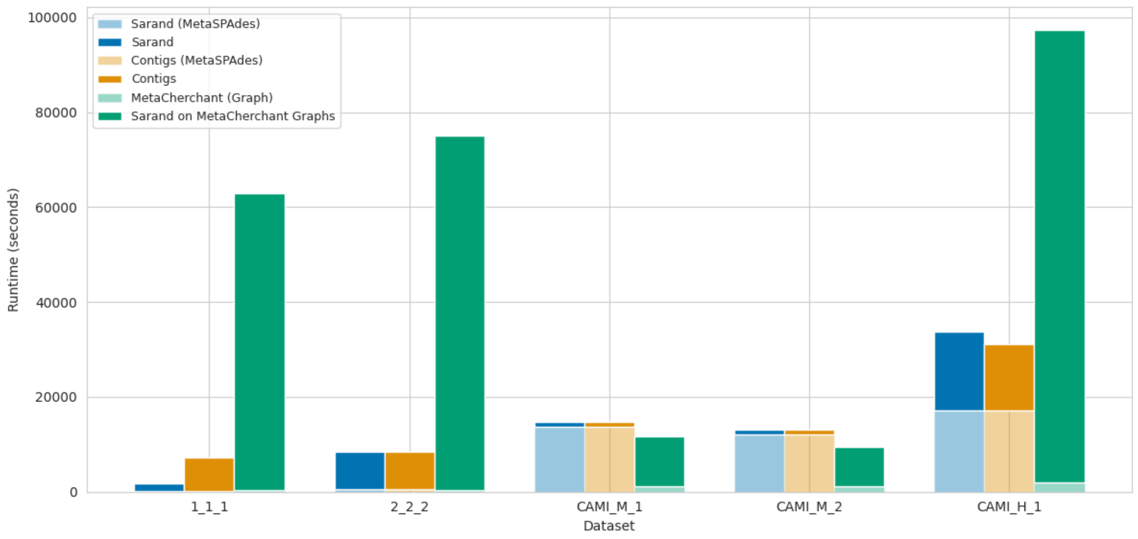

Figure S15: Comparison of runtime (in seconds) across all simulated datasets. Since both Sarand and the contig-based method require an assembler to first generate the assembly graph or contigs, the runtime of MetaSPAdes was included in the table. In addition, since MetaCherchant only generates subgraphs without generating neighbourhoods, we report the time taken to run MetaCherchant itself and generate the local graphs ("Metacherchant (Graph)"), as well as the time required to run Sarand (with a 2-minute time limit for extracting neighbourhood sequences of each AMR gene) for the MetaCherchant graphs to actually extract and annotate neighbourhood information ("Sarand on MetaCherchant Graphs").

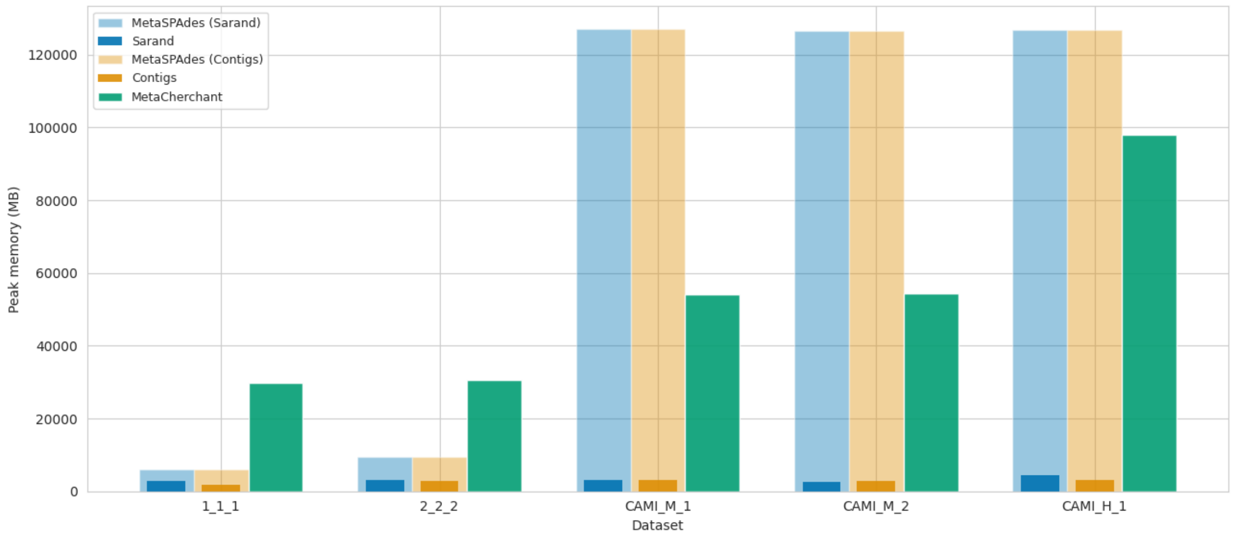

Figure S16: Comparison of peak memory consumption (in MB) across different datasets. For MetaCherchant, the numbers represent the peak memory usage during the execution of MetaCherchant to generate the local graphs. For Sarand and the contig-based method, we also report the peak memory usage during the MetaSPAdes run used to generate the assembly graph and contigs.
